# Impact of instrument error on the estimated prevalence of overweight and obesity in population-based surveys

**DOI:** 10.1186/1471-2458-13-146

**Published:** 2013-02-18

**Authors:** Anna Biehl, Ragnhild Hovengen, Haakon E Meyer, Jøran Hjelmesæth, Jørgen Meisfjord, Else-Karin Grøholt, Mathieu Roelants, Bjørn Heine Strand

**Affiliations:** 1 Division of Epidemiology, Norwegian Institute of Public Health, Nydalen, P.O. Box 4404, Oslo, 0403, Norway; 2 The Morbid Obesity Centre, Vestfold Hospital Trust, P.O. Box 2168, Tønsberg 3103, Norway; 3 Department of Community Medicine, Institute of Health and Society, University of Oslo, Blindern, P.O.Box 1130, Oslo 0318, Norway; 4 Laboratory of Anthropogenetics, Vrije Universiteit Brussel, Pleinlaan 2, Brussel 1050, Belgium; 5 Department of Public Health, Katholieke Universiteit Leuven, Kapucijnenvoer 35, Leuven 3000, Belgium

**Keywords:** Instrument error, Calibration, Anthropometry, Weights and measures, Obesity, Overweight, Epidemiology

## Abstract

**Background:**

The basis for this study is the fact that instrument error increases the variance of the distribution of body mass index (BMI). Combined with a defined cut-off value this may impact upon the estimated proportion of overweight and obesity. It is important to ensure high quality surveillance data in order to follow trends of estimated prevalence of overweight and obesity. The purpose of the study was to assess the impact of instrument error, due to uncalibrated scales and stadiometers, on prevalence estimates of overweight and obesity.

**Methods:**

Anthropometric measurements from a nationally representative sample were used; the Norwegian Child Growth study (NCG) of 3474 children. Each of the 127 participating schools received a reference weight and a reference length to determine the correction value. Correction value corresponds to instrument error and is the difference between the true value and the measured, uncorrected weight and height at local scales and stadiometers. Simulations were used to determine the expected implications of instrument errors. To systematically investigate this, the coefficient of variation (CV) of instrument error was used in the simulations and was increased successively.

**Results:**

Simulations showed that the estimated prevalence of overweight and obesity increased systematically with the size of instrument error when the mean instrument error was zero. The estimated prevalence was 16.4% with no instrument error and was, on average, overestimated by 0.5 percentage points based on observed variance of instrument error from the NCG-study. Further, the estimated prevalence was 16.7% with 1% CV of instrument error, and increased to 17.8%, 19.5% and 21.6% with 2%, 3% and 4% CV of instrument error, respectively.

**Conclusions:**

Failure to calibrate measuring instruments is likely to lead to overestimation of the prevalence of overweight and obesity in population-based surveys.

## Background

**Figure 1:**
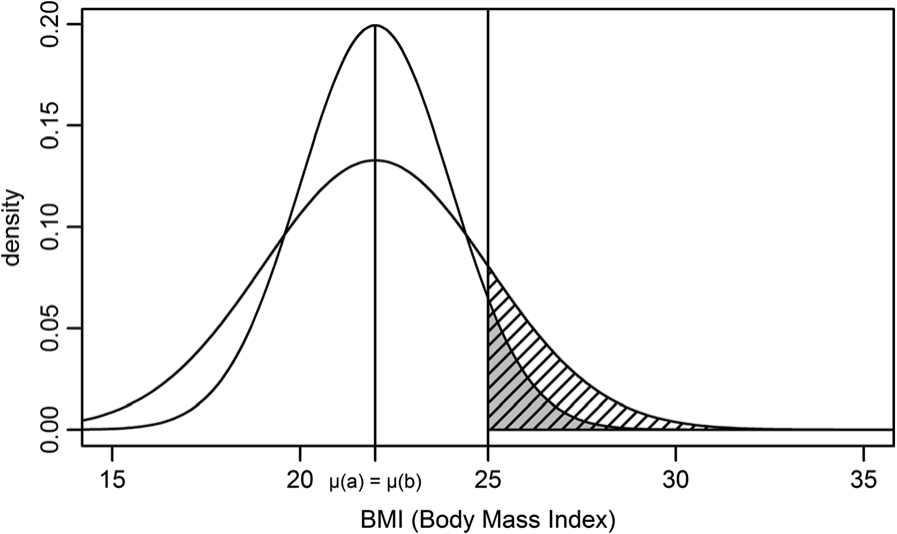
**Two hypothetical normal distributions of BMI with equal means but different variances; increased variance gives a lower and broader shape compared to a distribution with less variance.** Vertical line indicates cut-off value (BMI ≥ 25 kg/m^2^). The dark shaded area corresponds to the prevalence of overweight and obesity for the distribution with less variance, and the light shaded area corresponds to additional prevalence due to increased variance.

From a public health perspective there is a need for on-going surveillance and monitoring of the prevalence of overweight and obesity in the population [1-3]. It is important to ensure that surveillance data is of a high quality such that one can follow trends over time and compare results within and among countries. Anthropometric measurements, such as weight and height, are relatively inexpensive and easy to collect in population studies, but the validity of these measurements must be critically evaluated [4-8]. In epidemiological research and surveillance programs, the body mass index (BMI) (kg/m^2^) is widely used as a measure of adiposity. BMI cut-off values have been constructed to categorise individuals as underweight (BMI<18.5 kg/m^2^) [9], normal weight (18.5 kg/m^2^ ≤ BMI <25 kg/m^2^), overweight (25 kg/m^2^ ≤ BMI <30 kg/m^2^) or obese (BMI ≥30 kg/m^2^) [10]. Nevertheless, there is currently only a limited understanding of how using uncalibrated instruments impact upon BMI-based estimates of overweight and obesity. Only a few studies have considered how the inaccuracy of anthropometric measurements influences the interpretation of collected data [4,11]. For the present study, we have postulated that instrument error will not necessarily affect the mean height or mean weight, and thus mean BMI, but the variance in general will increase [7]. The height of a distribution curve is inversely proportional to the variance (SD) and instrument error will thus contribute to a flatter top to BMI distribution near the mean and more area in its tails [12] (Figure 1). Inaccurate height and/or weight measurements may therefore cause overestimation of the proportion above the cut-off value, i.e. the estimated prevalence of overweight and obesity.

### Measurement error and instrument error

The terminology used to describe anthropometric measurement error is not consistent [4,13]. Four frequently used concepts are precision, reliability, accuracy and validity [14]. The major sources of error associated with weight and height measurements of children are the observer, the child being measured (hydration and bladder contents, clothing, etc.), and the instrument used [5]. The precision and reliability of measurements refers to the extent to which repeated measurements give the same value, whereas accuracy and validity refer to how close a measurement is to its “true” value and is related both to the measurement technique as well as the measuring instrument [4,15]. Since a true value cannot be determined, in practice a conventional “true” value is used. The *accuracy* of a measuring instrument is the “*ability to give responses close to a true value*” [16:p.41]. In the present paper we focus on the accuracy of measuring instruments, which, in cases of inaccuracy will be referred to as *instrument error*. Components of measurement error associated with measurement technique will therefore be ignored. There are many reasons for the inaccuracy of measuring instruments, including overuse without maintenance or recalibration, incorrect usage and general wear and tear as a result of frequent transportation.

### Calibration vs. correction values

Measuring instruments should be calibrated according to a *standard* and adjusted in case of deviation [16]. Since population-based surveys often involve a large number of measurement sites, standardised calibration of all measuring instruments is an expensive and complicated procedure. As an alternative, the NCG-study developed a method where a *correction value* was determined for each instrument using a reference weight and a reference height. The correction value is equivalent to the instrument error and corresponds to the difference between the “true” value and the uncorrected value measured by an uncalibrated instrument. The term *reference* was used, since the method does not satisfy the requirements of a *standard* within metrology [16:p.46]. The procedure is described in detail below.

The main aim of the present study was to assess the degree to which instrument error, due to uncalibrated scales and stadiometers, impacts upon the overall estimated prevalence of overweight and obesity in population-based surveys. Growth data from a nationally representative sample and correction value data for all the instruments used in the study were utilised to illustrate the implications.

## Methods

To illustrate how instrument error affects the prevalence of overweight, we generated datasets from real data on weight and height from a sample of 8 year old children, to which simulated data with various degrees of instrument error were added. For each simulation a new prevalence estimate was produced, and after several simulations the mean and variance of the prevalence estimate could be calculated. Finally, to get an idea of how the size of the instrument error affects the prevalence estimate we successively increased the instrument error and re-ran the simulations.

### Study population

Anthropometric measurements were obtained in 2008 from a nationally representative sample of 3474 third grade pupils (~8 year olds) recruited in 127 primary schools as a part of *the Norwegian Child Growth Study (NCG)*. Participating schools were selected using a stratified two-stage sampling design following a protocol that was jointly developed by the WHO Regional Office for Europe and the participating Member States of the World Health Organization European Childhood Obesity Surveillance Initiative.

#### Ethical approval

The NCG-study was evaluated by the Regional Committee for Medical Research Ethics and approved by the Norwegian Data Inspectorate. Parents and guardians were informed about the study by letter beforehand and written informed consent was obtained from a parent or legal guardian via the school nurse prior to the study.

### Data collection

Prior to data collection all school nurses involved in the study received training in the standardised procedures of anthropometric measurement and the collection of correction values. Methods were explained and illustrated in a booklet specially developed for the NCG-study.

The measuring instruments used in this study were those already available on site, in that the types probably differed from one school to another. However, the usage and positioning of these instruments was standardised, e.g. scales had to be placed on a hard, horizontal floor.

### Anthropometric measurements

Body weight and height were measured according to standard procedures [17] and recorded to the nearest 0.1 kg and 0.1 cm respectively. Body Mass Index (BMI) was calculated as weight/height^2^ (kg/m^2^). Children were categorised as either under and normal weight (BMI < 25 kg/m^2^) and overweight or obese (BMI ≥ 25 kg/m^2^) using one cut-off value [10].

### Correction values

Correction values were collected in 2008 at the same time as anthropometric data collection. Each of the 127 schools received a reference weight and length that provided data from which to calculate the correction value for instruments at each school. The reference weights and lengths were within the range of the sample’s anthropometric measurements, i.e. a weight close to 28 kg for the scales and a length of 120 cm for the stadiometers.

The anthropometric measurements of the children were corrected post-hoc. The corrected measurements thus correspond to measurements taken by calibrated instruments and are assumed to be free of instrument error, although the instruments themselves were not calibrated.

#### Scales

A 25-l plastic container, filled with cold water, was used as a reference weight. Initially the containers were brought to the Norwegian Metrology Service and each container was numbered (1–127), filled with cold tap water and weighed on a calibrated scale. The reference weight of each container – the “true” weight - was registered in a protocol. The containers were then distributed to the participating schools and the school nurses were instructed to fill up the container with cold tap water until the last drop trickled over, cap it, weigh it once according to the standardised method and register the weight. Finally, we calculated the correction value (instrument error) for all 127 scales to be the difference between the “true” weight of the container and the uncorrected weight of the corresponding container measured at the school scale by the school nurse.

In cases where the measurements of the children were carried out over two days, the procedure of weighing the plastic container was not required to be repeated as long the scales were not moved.

#### Stadiometer

**Figure 2:**
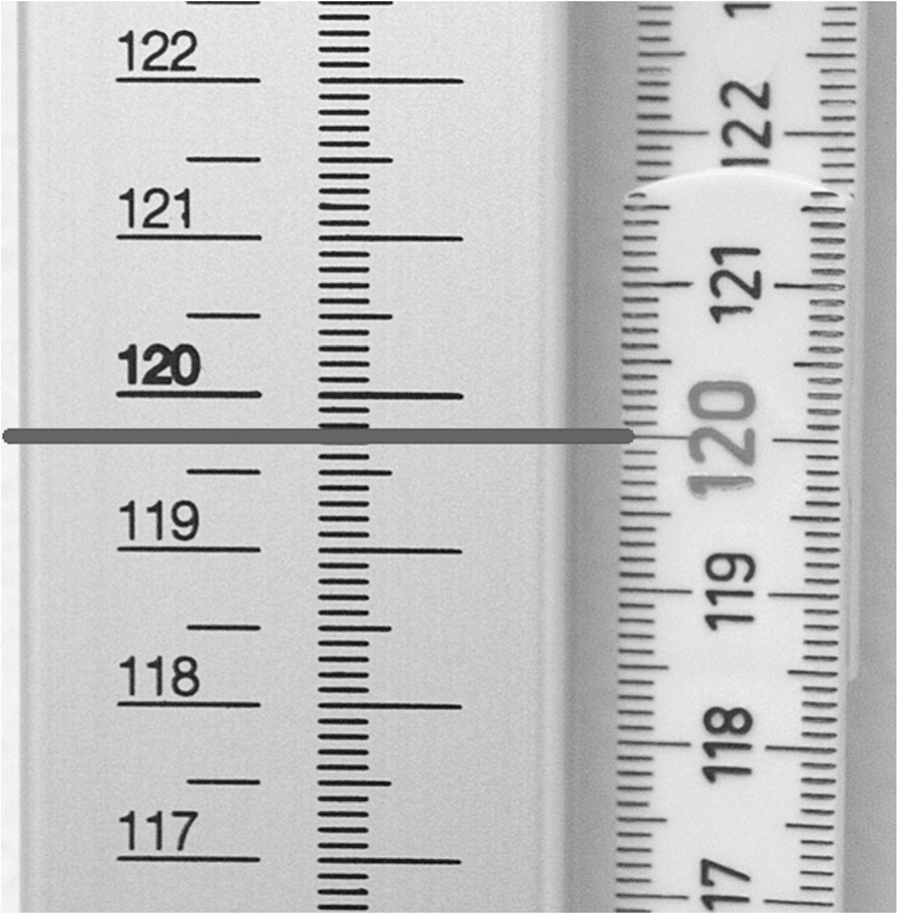
**Procedure to determine correction values in the NCG-study.** As part of the procedure to determine correction values in the NCG-study, the value of the stadiometer at each school (left) that corresponded to 120.0 cm of the reference length (right) was recorded. In practice, the folding rule was blacked out above and below 120 cm to avoid misunderstanding.

A wooden folding rule approved to EU class III [18] was used as a reference length. The folding rule was stabilised and straight before reading. Using the standardised method the school nurse recorded the value of the school stadiometer that corresponded to 120.0 cm on the reference length (Figure 2). The folding rule was blacked out above and below 120 cm to avoid misunderstanding. The correction value (instrument error) was calculated as the difference between 120.0 cm and the registered measure of the stadiometer at each school.

### Data analyses and simulation

To investigate the impact of instrument error on the estimated prevalence of overweight including obesity (BMI ≥25 kg/m^2^) we simulated random samples of corrected height- and weight data derived from the NCG-study and correction values (instrument error) were added in each simulation (n=1000). The coefficient of variation (CV) is an expression for the magnitude of instrument error and is useful to compare the variability in samples involving different measurements (weight in kilograms and height in metres).

Two different models were applied to systematically investigate how instrument errors affected the prevalence of overweight and obesity. To reduce the level of complexity we assumed the CV for both the scales and stadiometers to be equal in the *first model*. The CV of the correction value (instrument error) was increased gradually by 0.5 percentage points for both instruments according to the formula:

$$\mathrm{CV}\;\left(\%\right)=100^{x}\left(\mathrm{SD}/\text{mean}\right),$$

where *SD* is the standard deviation of the correction value (instrument error) and *mean* is the mean of the corrected height and weight values respectively. The corresponding SD of the correction value (instrument error) for scales and stadiometers was deducted for each CV value and used in the simulation model. The mean of error terms was set to zero. BMI was then calculated for each value of CV and the estimated prevalence of overweight and obesity was assessed (Table 1). The *second model* was run with the actual size of correction value (instrument error) from the NCG-study with differentiated CVs for scales and stadiometers and the mean was still assumed to be zero (Table 1).

**Table 1:** **An overview of the two models presenting the coefficient of variation (CV%), mean and SD of instrument error and the corresponding estimated prevalence of overweight and obesity (BMI ≥ 25 kg/m**^**2**^**) as mean, SD and the range (minimum-maximum values)**

	**Instrument error**				**Simulations**		
**CV(%)**	**Scales**		**Stadiometers**		**Runs (n)**	**Estimated prevalence of overweight and obesity (%)**	
	**Mean**	**SD**	**Mean**	**SD**		**Mean**	**SD (min-max)**
**Model I**							
0	0	0	0	0	1000	16.4	0 (16.4 – 16.4)
0.5	0	0.15	0	0.66	1000	16.5	0.17 (15.9 – 17.1)
1.0	0	0.29	0	1.32	1000	16.7	0.24 (16.0 – 17.5)
1.5	0	0.44	0	1.98	1000	17.2	0.28 (16.3 – 18.0)
2.0	0	0.58	0	2.64	1000	17.8	0.35 (16.7 – 19.2)
2.5	0	0.73	0	3.30	1000	18.6	0.39 (17.2 – 19.6)
3.0	0	0.87	0	3.96	1000	19.5	0.41 (18.1 – 20.7)
3.5	0	1.02	0	4.62	1000	20.5	0.46 (19.1 – 22.1)
4.0	0	1.16	0	5.28	1000	21.6	0.51 (19.5 – 23.2)
**Model II**							
2.1*/0.9**	0	0.64	0	1.11	1000	16.9	0.25 (15.8 – 17.7)

* CV of instrument error (%) of scales from the NCG-study.

**CV of instrument error (%) of stadiometers from the NCG-study.

All simulations were programmed in STATA 11.

## Results

### Correction values

On average it was observed that the instruments in the NCG-study underestimated weight and height measurements. The correction values of scales ranged from −3.0 to +1.7 kg, mean (SD): –0.14 (0.64) and from −3.5 to +4.0 cm, mean (SD): –0.07 (1.11) for stadiometers. In the NCG-study the CV of instrument error of the scales and stadiometers was 2.1% and 0.9%, respectively. This indicates that the CV of instrument error was at least twice as large for scales compared to stadiometers.

### Estimated prevalence

**Figure 3:**
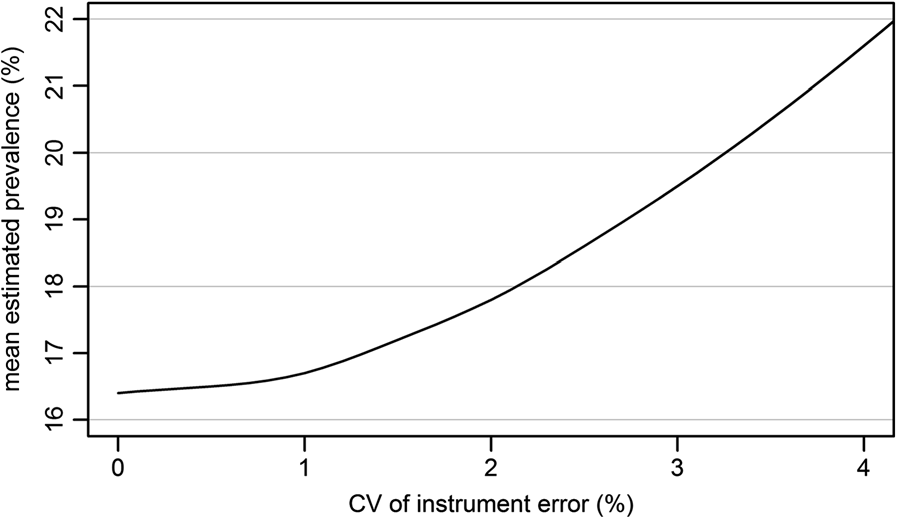
**The mean estimated prevalence (%) of overweight and obesity (BMI ≥ 25 kg/m**^**2**^**) in relation to the coefficient of variation (CV) of instrument error (%).**

Simulations showed that the estimated prevalence of overweight and obesity changed systematically with increased instrument error (Figure 3). The estimated prevalence of overweight and obesity was 16.4% with no instrument error, corresponding to measurements taken by calibrated instruments, with the mean of error term set to zero. The mean of the estimated prevalence increased to 16.7% with 1% CV of instrument error, and to 17.8%, 19.5% and 21.6% with 2%, 3% and 4% CV of instrument error, respectively (Table 1).

The prevalence was underestimated in a minority of the simulated samples. When the CV of instrument error was 1%, about 1 in 12 of the simulated samples underestimated the true prevalence. When the CV was 1.5% this happened only 1 in 200 of the simulated samples, and when the CV was 2% or greater no simulated samples underestimated the true prevalence.

In the *second model*, using the actual size of the CV of instrument error of the NCG-study, uncorrected estimates showed an average overestimation corresponding to 0.5 percentage points of the prevalence of overweight and obesity among 8 year-olds (Table 1).

## Discussion

In this study we found that instrument error due to uncalibrated scales and stadiometers, combined with cut-off based classification systems, can lead to minor but systematic overestimation of the prevalence of overweight and obesity in a nationally representative sample.

To the best of our knowledge, this is the first study to show how increased variance of anthropometric measurements can affect outcome measures in obesity surveillance. Previous studies have considered the accuracy of anthropometric measurements, but did not evaluate the impact on the estimated prevalence of overweight including obesity (BMI ≥25 kg/m^2^) [7]. It has been reported that scales in healthcare settings did not have higher accuracy than scales in fitness- or weight loss centres [19]. Furthermore, beam-balance scales are more accurate than scales with electronic mechanisms, whilst bathroom-type scales with a spring mechanism are least accurate [11]. These findings indicate that it is possible to increase accuracy by ensuring the school health service has good quality instruments.

Our findings are particularly relevant for population-based studies and surveillance programs that maximise the use of existing resources, such as instruments in the school health service. It is important to consider and balance accuracy with feasibility [20]. We thus developed a simple yet effective procedure for the NCG-study in order to obtain correction values for instruments at each school. This need was clearly demonstrated by the wide range of instrument error (4.7 kg and 7.5 cm) observed in this study from 127 scales and stadiometers. Valid data were collected with limited costs. We also found that, on average, instruments in the NCG-study slightly underestimated both weight (mean: –0.14 kg) and height (mean: –0.07 cm) measurements. However, this information was not used in the current analyses since the aim of this study was solely to assess the effect of instrument error variation. The average of the error terms was therefore set to zero in the simulations. If the mean error term was not set to zero, but rather set to the values observed in the NCG-study, the entire distribution would have shifted to the left due to the negative means, whilst the effect of “heavy tails” demonstrated in this study would have been equalised or toned down.

A possible limitation of the approach adopted in the NCG-study, i.e. adjusting for the correction value, is that it may only be valid for the specific point on the measuring-scale that corresponds to the reference weight and length value. To ensure the collection of the correction value on the appropriate part of the measuring-scale in weighing scales, a reference weight was chosen within the range of value of our target population. For stadiometers corrected at one point, measurements are likely to be correct along the whole measuring-scale.

According to the procedures, the plastic containers used to collect correction values were only measured once. Duplicate measurements would have increased the reliability, but optimisation and feasibility must be considered. Overly complicated procedures would undoubtedly increase the risk of dropout. In the present study, no schools dropped out. At the majority of schools, the data collection was completed within one day.

Only a single average cut-off value for overweight and obesity was used for the entire sample of 8–9 year old girls and boys in the simulations, and not age- and sex specific cut-off values as recommended by the IOTF [10]. This was done deliberately, in order to simplify the analyses. The expressed aim of the study was to explore the phenomenon of increased variance rather than to present correct estimates of the prevalence of overweight including obesity. For the same reason, the two-stage sampling methodology was not taken into account in the simulation analysis.

The rationale behind running the simulations according to two different models was to illustrate, in the first model, that an increasing instrument error will systematically impact upon the estimated proportion of overweight and obesity in surveys. The second model contained the actual CVs of instrument error for scales and stadiometers derived from the NCG-study and serves as a realistic example.

In the literature it is often stated that instruments are calibrated prior to data collection, without giving detailed calibration procedures. It has been claimed that once instruments are installed and calibrated, error due to the instruments is negligible [5]. Our findings suggest the contrary. Indeed, the impetus for this paper stems from the finding that old flooring had been removed from a school health office without adjustments to the wall-mounted stadiometer and with subsequent inaccurate height measurements. It shows that even when measuring instruments are calibrated upon acquisition and installment they can become uncalibrated, underlining the need for procedures for regular maintenance of anthropometric instruments. Generally, measuring instruments are bought calibrated, but due to lack of awareness, they may never be maintained or recalibrated after years of use.

## Conclusions

To the best of our knowledge, this is the first study to demonstrate that instrument error will systematically increase the estimated prevalence of overweight and obesity. This study emphasises the need for regular maintenance or recalibration of instruments in order to reduce instrument error and to obtain more accurate estimates in populations-based surveys. Although the present analysis is limited to the use of inaccurate height and weight measuring instruments, the outlined principle is generic and can be applied to measurement error in general.

## Competing interests

The authors declare that they have no competing interests.

## Authors' contributions

RH had the original idea. AB and BHS did most of the statistical analyses and prepared the first draft. All authors participated in the discussion, interpretation of the results and contributed in writing the manuscript and had final approval of the submitted and published version.

## Pre-publication history

The pre-publication history for this paper can be accessed here:

http://www.biomedcentral.com/1471-2458/13/146/prepub
